# 185 Real-World Uptake and Impact of Maternal Respiratory Syncytial Virus (RSV) Vaccination and Infant Nirsevimab in Infants ? 6 Months

**DOI:** 10.1017/ash.2026.10439

**Published:** 2026-06-23

**Authors:** Jacob Pierce, Jacci Thomas

**Affiliations:** 1 Brody School of Medicine at East Carolina University; 2 ECU Health

## Abstract

**Background:** Hospital acquired infections are known to increase hospital stays and increase morbidity and mortality. Historically, Methicillin-resistance Staphylococcus aureus (MRSA) screening and contact precautions have been the primary strategy to prevent the spread of MRSA in healthcare systems. Increasing evidence supports the role for MRSA decolonization to decrease the spread of this important pathogen. The COVID-19 pandemic and related personal protective equipment conservation has led to many programs re-examining contact precautions for MRSA. **Methods:** In February of 2024 MRSA screening was discontinued for new admissions and an MRSA decolonization protocol was implemented for patients at high risk for MRSA infection as defined in the SHEA compendium for reducing MRSA infections. Decolonization included nasal Mupirocin 2% ointment and 2% Chlorhexidine Gluconate (CHG) bathing for five days in the adult inpatient population. High risk patients targeted for this intervention included patients transferred from another healthcare facility, admitted from home health, on dialysis, prior history of MRSA infection or those with central venous catheters. Contact isolation precautions for MRSA colonization were discontinued but maintained for active infection with MRSA. **Results:** After implementation, MRSA nares screening tests declined by 24.4% while utilization of mupirocin increased 5.85 times the pre-implementation average. In the twelve months prior to the intervention there were 221.1 new MRSA infections per 1000 inpatient admissions and in the 12 months following intervention there were 155.4 new MRSA infections per 1000 inpatient admissions. The rate of MRSA infections had been increasing prior to intervention and the rate of increase decreased by 54.8% after implementation. We estimated a cost savings of $194,745 if all patients that were previously screened were instead decolonized. We additionally noted an approximate 18% reduction in isolation gown use resulting an additional estimated annual cost savings of $115,369 per year if sustained. **Conclusions:** The implementation of a MRSA decolonization program resulted in significant cost savings without any associated increase in clinical MRSA cultures. The program appeared to have far greater buy-in than the previous screen and isolate program despite having similar patient selection criteria. This may reflect a greater perceived importance of screening relative to a treatment strategy. A decolonization strategy was found to be effective at decreasing the rate of new MRSA infections even with the removal of isolation precautions for colonization. Low compliance with isolation precautions for colonization are thought to be a contributing factor in the limited efficacy of a screen and isolate strategy.

